# The impact on lateral wall fractures by a sliding hip screw device in trochanteric fractures: a biomechanical study

**DOI:** 10.1302/2633-1462.65.BJO-2024-0266.R1

**Published:** 2025-05-29

**Authors:** Magnus Høgevold, Carl E. Alm, Bryan J. Wright, Lydia Ragan, Frede Frihagen, Stephan M. H. Röhrl, Jan E. Madsen, Jan E. Brattgjerd

**Affiliations:** 1 Department of Orthopaedic Surgery, Diakonhjemmet Hospital, Oslo, Norway; 2 Institute of Clinical Medicine, University of Oslo, Oslo, Norway; 3 Division of Orthopaedic Surgery, Department of Orthopaedic Surgery, Oslo University Hospital, Oslo, Norway; 4 Aleris Health, Oslo, Norway; 5 Division of Anatomy, Institute of Basic Medical Sciences, University of Oslo, Oslo, Norway; 6 Department of Rehabilitation Science and Health Technology, Faculty of Health Sciences, Oslo Metropolitan University, Oslo, Norway; 7 Kalnes Hospital, Fredrikstad, Norway

**Keywords:** Biomechanical study, Cadaveric bone, Osteotomies, Trochanteric fracture, Lateral wall, Lesser trochanter, Sliding hip screw, trochanteric fractures, sliding hip screw, fractures, lesser trochanter, Orthopaedic Trauma, stiffness, femora, Lag screw, femoral head

## Abstract

**Aims:**

Trochanteric fractures with a reduced lateral wall thickness and a detached lesser trochanter are unstable. These characteristics can lead to failure through a lateral wall fracture after fixation with a sliding hip screw device (SHS). However, the precise mechanism by which these characteristics contribute to lateral wall fractures remains unclear. Accordingly, we examined the impact on failure by incremental decrease of both lateral wall thickness and lesser trochanter attachment in trochanteric fracture fixation using a SHS ex vivo.

**Methods:**

We tested 14 pairs of embalmed femora, randomly assigned to four osteotomy groups, each representing a specific 31A1 or 31A2 fracture pattern according to the AO/Orthopaedic Trauma Association (OTA) classification. After fixation with a SHS, the specimens underwent quasi-static and dynamic axial compression until failure. Outcome measures included stiffness, deformation, load to failure, and failure pattern following a set protocol.

**Results:**

We found lateral wall fractures in ten out of 28 specimens. Specimens with a thin lateral wall and a detached lesser trochanter exhibited both a significantly higher rate of lateral wall fractures (5/7 vs 5/21, p = 0.023) and a lower load to failure than specimens with only one or none of these characteristics (1,673 N (SD 810) vs 2,922 N (SD 897), p = 0.003). The specimens with a detached lesser trochanter themselves demonstrated a significantly higher rate of lateral wall fractures than those with an attached lesser trochanter (9/14 vs 1/14, p = 0.004).

**Conclusion:**

Our findings emphasize the role of a detached lesser trochanter in initiating lateral wall fractures, with a reduction in load to failure when combined with reduced lateral wall thickness. Biomechanically, this suggests a failure mechanism whereby placing a load-sharing implant could overload a fragile lateral wall in unstable trochanteric fractures.

Cite this article: *Bone Jt Open* 2025;6(5):582–589.

## Introduction

In trochanteric fractures, mechanical failures are a considerable challenge. A 3% to 11% reoperation rate has been reported in patients with these fractures after treatment with a sliding hip screw device (SHS).^1^ Mechanical complications are the primary cause of these reoperations, involving peri-implant fractures as in the lateral wall, lag-screw cut-out, and implant failure. Ultimately, complications as a lateral wall fracture may lead to prolonged rehabilitation and nonunions, requiring revision surgery.^1-4^

The mechanical failure patterns in trochanteric fractures are typical. A lateral wall fracture initiates at the lag-screw entry-point, with loss of the lateral buttress, shaft medialization, and lag-screw lateralization.^4^ The lag-screw cutting-out from the femoral head leads to varus of the neck-shaft angle.^5^ Implant failure includes the less defined hardware breakdown.^1^ This may be breakage of the side plate, back-out of the lag-screw, or pull-out of the side plate screws.

With these patterns of mechanical failure, ex vivo testing of trochanteric fractures has an advantage by allowing intentional overloading.^6^ Both a reduced lateral wall thickness (LWT) mm and a detached lesser trochanter have been recognized to increase risk of reoperation. Up to a 35% incidence of lateral wall fractures, potentially leading to treatment failure, has been reported with these characteristics after fixation with a SHS.^2,3^ The agreement on importance of these characteristics is reflected by their use as stability criteria in different versions of the AO/Orthopaedic Trauma Association (OTA) classification.^7-9^

Recent in vitro studies confirm that both LWT and lesser trochanter detachment contribute to the stability of trochanteric fixation.^10,11^ Yet, the mechanism by which they act together to induce a lateral wall fracture remains unclear, and there is a need for studies to explore the stepwise increase of assumed instability affecting these structures. This approach may elaborate our understanding of trochanteric fracture stability. It may also guide the classification and management of these fractures.

Our aim was to investigate how both LWT and detachment of the lesser trochanter contribute to failure due to a lateral wall fracture in trochanteric fractures instrumented by a SHS. We hypothesized that both these characteristics were necessary for reducing resistance to lateral wall fractures. We tested this hypothesis by evaluating the incremental decrease of trochanteric stability in an ex vivo model.

The Regional Committee for Medical and Health Research Ethics Norway approved the present study, with reference number 2011/1479.

## Methods

### Preparations

We included 14 pairs of femora from Caucasian cadavers. Six pairs were from females, and eight were from males, with a mean age of 81 years (61 to 94). Embalmed femora were used, following a standardized preparation routine with reported well-conserved mechanical properties and mineral content.^12^ After removal of soft-tissues, the femora were kept moist until testing.

To match pairs based on bone mineral density, we performed a CT scan (Siemens SOMATOM Definition Edge; Siemens Healthcare, Germany). Bone mineral density was measured in the femoral head.^13^ These scans were also used to rule out the presence of bony abnormalities.

We used a randomization website to allocate femora at random into two paired osteotomy groups (Groups A and B or Groups C and D). This partially paired design enabled a paired comparison between Groups A and B and between Groups C and D, and otherwise matched comparisons (Table I).

**Fig. 1:**
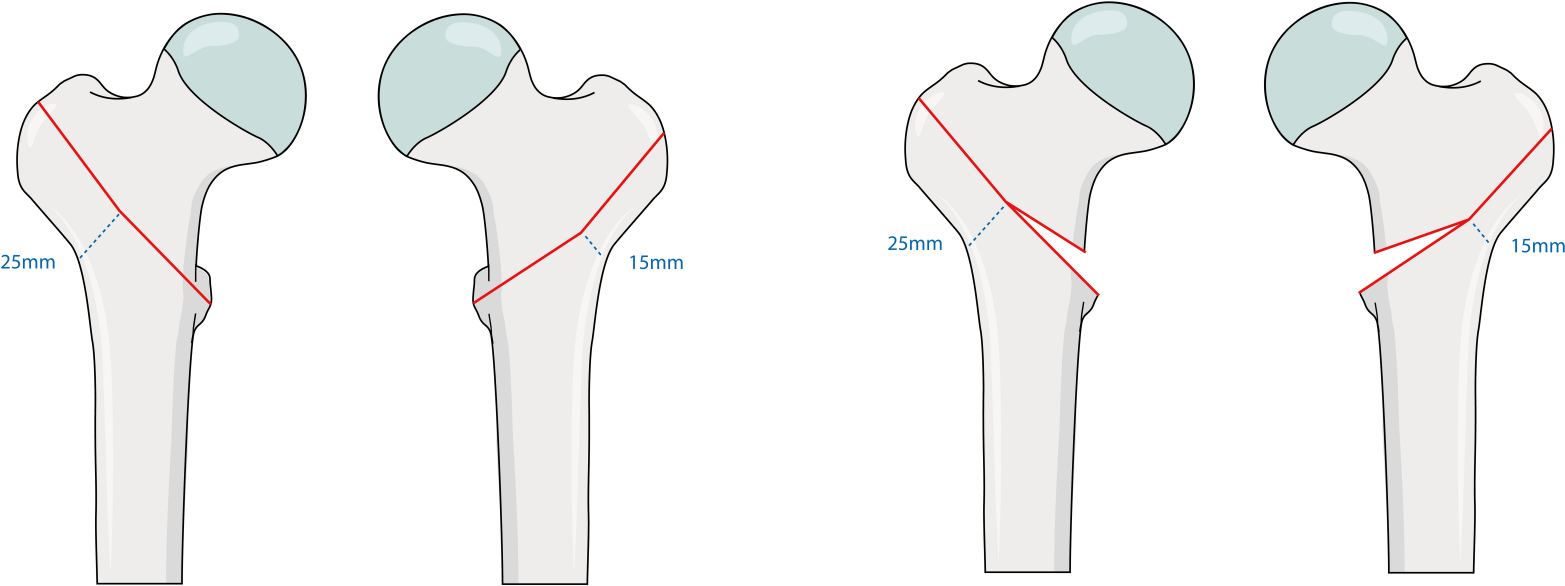
The four trochanteric osteotomy groups A to D. Lateral wall thickness (LWT) is illustrated. Group A: a two-part osteotomy with a LWT of 25 mm, defined as AO/Orthopaedic Trauma Association (OTA) Type 31A1. Group B: a corresponding osteotomy as in Group A, defined by a LWT of 15 mm as AO/OTA Type 31A2. Group C: an intermediate wedge fragment of the lesser trochanter and a LWT of 25 mm as AO/OTA Type 31A1. Group D: a corresponding osteotomy as in Group C, defined by a LWT of 15 mm as AO/OTA Type 31A2.

To evaluate the stepwise impact of fracture stability, we selected a 10 mm difference in LWT combined with a complete or osteotomized lesser trochanter.^10,11^ Four different osteotomies, simulating trochanteric A1 and A2 fractures, as defined in the 2018 AO/OTA classification, were performed (Figure 1).^7^ In Group A, a two-part osteotomy with a LWT of 25 mm was cut (A1). Group B simulated the corresponding osteotomy, defined by a LWT of 15 mm as an A2 fracture. In Group C, an intermediate wedge fragment of the lesser trochanter was removed next to a LWT of 25 mm (A1). The intermediate fragment of the lesser trochanter was also removed in Group D, with a LWT of 15 mm (A2).

**Table I.** Comparison of the four groups.

Groups	Mean age, yrs (SD)	Sex, female:male	Mean bone mineral density, HU (SD)
A (thick wall, intact lesser trochanter)	81.4 (11.6)	3:4	181 (53)
B (thin wall, intact lesser trochanter)	81.4 (11.6)	3:4	190 (95)
C (thick wall, no lesser trochanter)	81.1 (9.3)	3:4	198 (80)
D (thin wall, no lesser trochanter))	81.1 (9.3)	3:4	221 (67)

HU, Hounsfield units.

Concerning the technique of lag screw placement, two surgeons (MH, CEA) predrilled all specimens to ensure an anatomical reduction.^5^ A 2.5 mm guide wire in the 135° angled guide was used to aim for a central position in the femoral head, both frontally and laterally. Subsequently, drilling for a lag screw with a 12.5 mm thread diameter and a four-hole 135° Dynamic Hip Screw Locking Compression Plate (DePuy Synthes, USA) was carried out in each specimen.

During the osteotomies, we measured LWT along the drilled lag screw channel.^2,7^ The osteotomies were cut parallel to the wall, down to this channel, and angled through the most prominent part of the lesser trochanter in all specimens. An additional cut through the proximal end of the lesser trochanter was chosen to simulate a detached lesser trochanter by intermediate fragmentation in Group C and Group D.^7^ This extra cut generated a gap osteotomy after the removal of a wedge involving the lesser trochanter.

For fixation of the osteotomies with a SHS device, we used a wrench for the one-step inserting technique for the lag screw and tightening of the self-tapping 4.5 mm cortical screws with an AO screw driver, as performed clinically. The lag screw’s tip apex distance (TAD) of less than 25 mm was confirmed using fluoroscopy.^14^ The resulting mean TAD was 12 mm (6 to 18), ensuring seven clinically realistic and standardized specimens in each group.

### Mechanical testing

**Fig. 2:**
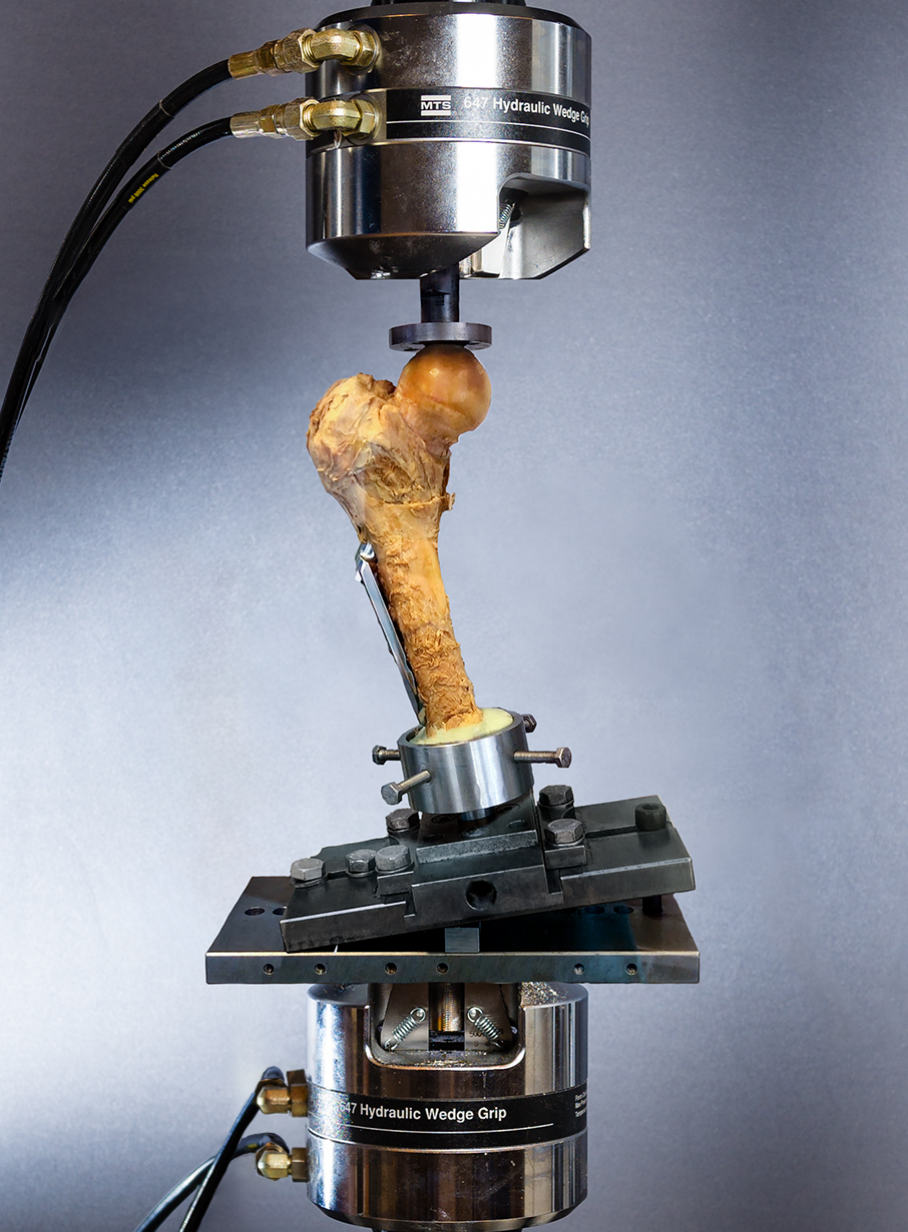
The test set-up. An instrumented specimen cemented distally with a flat metal piston on top, mounted in a material testing machine from MTS Systems (USA).

We mounted a 15 cm long proximal femora rigidly in a neutral, upright position by distal cementing (Biomet Bone Cement; Zimmer Biomet, USA) (Figure 2).^10^ A flat metal piston on the actuator of our material testing machine transmitted load at the femoral head (MiniBionix 858; MTS Systems, USA). A load cell applied the axial compressive load (capacity 10 kN; resolution 1 N; displacement 0.001 mm; accuracy < 0.5%; sampling rate 100 Hz). A computer recorded load and displacement of the piston (MTS FlexTest 40 with Station Manager, USA).

To mimic the angle of hip joint reaction force during stumbling and walking, we tilted specimens into 20° adduction.^15^ First, an axial preload was applied (30 N).^6^ Then, quasi-static, non-destructive loading was performed (rate 200 N/s, three times, load 250 N).^6^ To simulate postoperative weightbearing with a relevant number of steps until consolidation, dynamic loading was used,^16,17^ with a sinusoidal loading pattern (rate 2 Hz, cycles 10,000, maximum load 750 N). To simulate the worst-case scenario with a reaction force reaching eight times body-weight during stumbling,^15^ a final quasi-static compressive load to failure test was conducted (rate 250 N/s, maximum load 10 kN).^6^

### System of measurements

Outcomes were axial stiffness, deformation, and load to failure with failure pattern identification. The stiffness of each specimen was calculated as the average from the initial quasi-static compressions. Deformation was measured after dynamic testing. Load to failure was defined as the maximum force until failure. Failure was defined as an abrupt increase in deformation until 10 mm, which terminated the test. Failure patterns were described as they occurred. Outcome in specimens were averaged within groups and expressed as means or rates.

We calculated the sample size using the rate of lateral wall fractures as the primary outcome. This calculation was based on a 100% reduction in the rate of lateral wall fractures, as previously reported by Hsu et al;^2^ in their study comparing 111 patients with trochanteric fractures involving a detached lesser trochanter, they observed lateral wall fractures in all 39 patients with a mean LWT of 23 mm, while no such fractures occurred in 72 patients with a mean LWT of 18 mm. To test the null hypothesis of no effect on lateral wall fractures by reducing LWT in osteotomies with a detached lesser trochanter, seven femora were enough in each of these groups (Groups C and D). This sample size ensured a power of 90% in all comparisons with a significance level of p < 0.05.

### Statistical analysis

For statistical analyses, we used SPSS v. 29 (IBM, USA). The data were assessed for normality using the Shapiro-Wilk test. Comparisons between multiple groups were analyzed using McNemar’s test for categorical variables. One-way analyses of variance with post-hoc Bonferroni correction were used for continuous variables. The level of significance was set at p < 0.05.

## Results

**Fig. 3:**
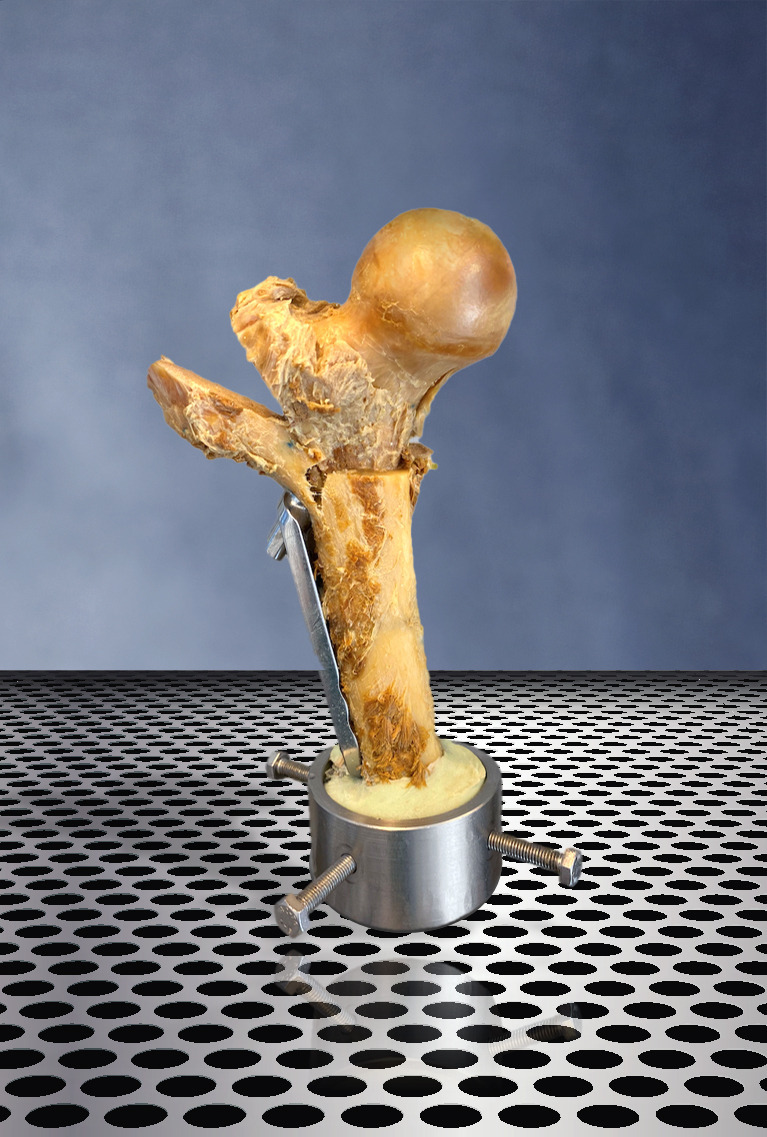
The lateral wall fracture (LWF). A specimen demonstrating a failure pattern of a LWF, originating at the level of the lag screw drilling site.

Failure mode was a lateral wall fracture in ten out of 28 specimens (Figure 3), with femoral head cut-outs, implant failures, or a femoral neck fracture in the remaining (Table II). The mean load to failure with lateral wall fractures was 938 N lower than with the other failure patterns (95% CI -1,693 to -183, p = 0.017)

**Table II.** Biomechanical performance of groups with comparisons.

Groups	Stiffness (N/mm)	Deformation (mm)	Load to failure (kN)	Lateral wall fracture (n/N)	Lag screw bending (n/N)	Neck fracture (n/N)	Cut-out (n/N)
A (thick wall, intact lesser trochanter)	228 (156)	2.0 (1.5)	2.86 (0.62)	0/7	1/7	1/7	5/7
B (thin wall, intact lesser trochanter)	235 (115)	2.8 (2.4)	3.08 (1.04)*	1/7	2/7	0/7	4/7
C (thick wall, no lesser trochanter)	160 (68)	2.6 (1.3)	2.83 (1.09)	4/7	2/7	0/7	1/7
D (thin wall, no lesser trochanter))	141 (62)	3.6 (3.0)	1.67 (0.81)*†	5/7§	2/7	0/7	0/7
A + B (intact lesser trochanter)	231 (132)	2.4 (2.0)	2.97 (0.83)	1/14 ¶	3/14	1/14	9/14
C + D (no lesser trochanter)	150 (63)	3.1 (2.3)	2.25 (1.10)	9/14¶	4/14	0/14	1/14
A + C (thick wall)	194 (121)	2.3 (1.4)	2.84 (0.85)	4/14	3/14	1/14	6/14
B + D (thin wall)	188 (101)	3.2 (2.6)	2.37 (1.15)	6/14	4/14	0/14	4/14
A to C (not D)	208 (118)	2.5 (1.8)	2.92 (0.90)†	5/21§	5/21	1/21	10/21
Lateral wall fractures	170 (83)	3.2 (2.8)	2.01 (1.18)‡	10/10			
Other failure patterns	202 (122)	2.5 (1.7)	2.94 (0.77)‡	0/10	7/18	1/18	10/18

Values are shown as mean (SD).

* Lower mean load to failure in Group D than in Group B (p < 0.05)

† Lower mean load to failure in Group D than in the other groups combined (p < 0.05)

‡ Lower mean load to failure in specimens with lateral wall fracture than in specimens with other failure patterns (p < 0.05)

§ Higher rate of lateral wall fractures in Group D than in the other groups combined (p < 0.05)

¶ Higher rate of lateral wall fractures in the groups with deteched lesser trochanter than in groups with intact lesser trochanter (p < 0.05)

The combination of a thin lateral wall and detachment of the lesser trochanter was observed to impact both the type of failure pattern and the load at which these failures occurred. Osteotomies with both these characteristics showed a 48% higher rate of lateral wall fractures than those with one or none of them (5/7 vs 5/21, p = 0.023) (Group D vs Groups A, B, and C). Also, a difference in mean load to failure was found between the same groups, showing a 1,249 N (43%) decrease in load to failure with both characteristics (95% CI -2,036 to -461, p = 0.003) (Group D vs Groups A, B, and C).

Detachment of the lesser trochanter itself significantly impacted both failure patterns and load to failure. A 57% higher rate of lateral wall fractures was detected in osteotomies with a detached lesser trochanter than in those with an attached one (9/14 vs 1/14, p = 0.004) (Groups C and D vs Groups A and B). Also, a lower rate of cut-outs was found with a detached lesser trochanter than if it was attached (1/14 vs 9/14) (Groups C and D vs Groups A and B). In addition, mean load to failure was 1,403 N (46%) lower in osteotomies with both characteristics compared to those with only a reduced LWT (Group D vs Group B) (95% CI -2,488 to -319, p = 0.048).

No other significant differences were identified, neither in terms of an impact of LWT nor in stiffness and deformation outcomes.

## Discussion

Our aim was to analyze the contribution of LWT and lesser trochanter detachment to failure due to a lateral wall fracture in trochanteric fixation using a SHS ex vivo. We found a high rate of lateral wall fractures, which occurred at a lower load than the other failure patterns. The rate of lateral wall fractures ranged from one out of 14 (7%) in cases with an intact lesser trochanter (Groups A and B), to nine out of 14 (64%) in cases with a detached lesser trochanter (Groups C and D), with five out of seven (71%) of those in Group D also having a thin lateral wall. Both a reduced LWT and a detached lesser trochanter were necessary to reduce resistance to failure by a lateral wall fracture (Groups A to C vs Group D and Group B vs Group D). Lesser trochanter detachment itself also influenced the type of failure pattern (Groups A and B vs Groups C and D).

In the review of the literature on postoperative lateral wall fractures, the role of fracture characteristics in predicting these failures is highlighted. Hsu et al^2^ performed a retrospective study in 208 patients with a SHS as fixation of trochanteric fractures. They found a higher incidence of lateral wall fracture in fractures in 111 patients with a detached lesser trochanter (35% vs 3%, p < 0.001). In these patients, the rate of lateral wall fractures was significantly higher (100%) in the 39 individuals with an additional reduction in LWT to 18 mm, compared to 0% in the 72 patients with a mean LWT of 23 mm (p < 0.001). These clinical failure rates, along with the associated risk factors, closely align with the results observed in our study groups, experimentally validating the role of these characteristics. Therefore, the present study provides biomechanical evidence of the increased susceptibility to a lateral wall fracture using a SHS in patients with a trochanteric fracture and a detached lesser trochanter, particularly due to the association between a diminished LWT and a lower load to failure.

**Fig. 4:**
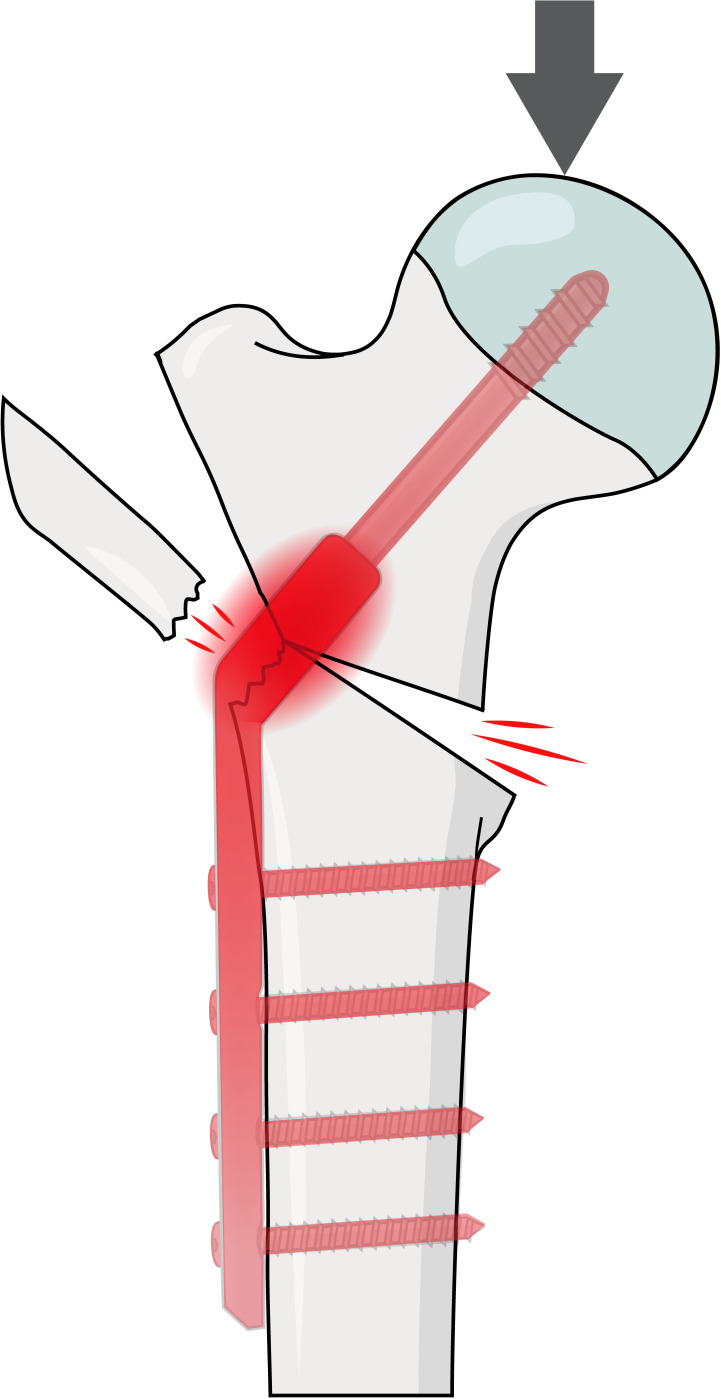
The failure mechanism of a lateral wall fracture. In trochanteric fractures, a gap osteotomy involving the lesser trochanter reduces absorption of compression (indicated by red lines medially), which increases load bearing on the implant (indicated by the implant coloured red). This heightened load causes stress to concentrate at the junction of the barrel and side plate. When the lateral wall thickness is low, this increased stress can overload the lateral wall (indicated by red lines laterally), leading to a lateral wall fracture. This mechanism of failure explains a fracture of the lateral wall as both a fracture and implant.

There is a paucity of documentation on the exact failure mechanism of the lateral wall. While recent biomechanical studies have analyzed the individual contribution of fracture characteristics to stability of trochanteric fractures, their combined contribution is less understood. Marmor et al^10^ investigated the impact of lesser trochanter osteotomies on implant load bearing by using strain gauges ex vivo. In their study, load bearing by a SHS increased gradually from 8% in the intact state to 80% with an extended gap osteotomy (p < 0.001). Fan et al^11^ assessed the effect of decreasing LWT on von Mises stress in a finite element analysis. They reported that proximal fragment stress increased by 81% when the LWT was reduced from 30 mm to 10 mm, peaking within the SHS at the junction of the barrel and side plate. The increased implant load-bearing with a SHS due to a reduced medial cortical buttress,^10^ increased stress in the proximal fragment, peaking at the lag screw drilling site laterally, which developed into a lateral wall fracture,^4^ correspond well with our findings. Furthermore, our main results indicate that the mechanism of a lateral wall fracture was initiated at a low loading level with a load-sharing implant (Figure 4). In group D, this magnitude was below 260% body weight, which equals the hip’s peak joint reaction force during normal walking (assuming a body weight of 75 kg).^18^ This finding also aligns well with the results of a retrospective study by Palm et al.^3^ They observed that 74% out of 46 lateral wall fractures in 214 patients with trochanteric fractures treated with a SHS occurred during the operative procedure itself. On the contrary, with the medial buttress and/or lateral wall remaining intact, reduced implant load-bearing^10^ and lateral stress^11^ explain the lower rate of lateral wall fractures (Groups A to C vs Group D). In these groups, the medial structures failed before the lateral, resulting in a failure pattern characterized by either lag screw cut-out or bending at a higher LTF. Moreover, migration profiles of trochanteric fractures with a detached lesser trochanter were analyzed in a recent radiostereometric study.^19^ The finding of similar migration profiles after using a SHS, with or without an additional support plate reinforcing the lateral wall,^19^ agree with our findings of no impact on stiffness and deformation with the characteristics in the present study. We interpret that a difference in non-destructive parameters was prevented by increased implant load bearing, compensating reduced fracture stability. In failure testing, it seems that the presence of both characteristics produced a threshold phenomenon. This argument is supported by no convincing difference in stiffness or deformation, which was replaced by a lateral wall fracture if fracture stability was sufficiently reduced in the current study. We suggest efforts to restore stability in the least stable fractures. To provide a more stable construct, both reduction of the lesser trochanter and adding lateral support with a trochanteric stabilizing plate may be beneficial.^20,21^ This recommendation is based on a higher load to failure and less frequent lateral wall fractures in the more stable osteotomies than in the unstable ones (Groups A to C vs Group D, and Groups A and B vs Groups C and D). Hence, the present study confirms the biomechanical role of a reduced load to induce a lateral wall fracture with both characteristics in trochanteric fixation using a SHS.^3^ Also, our study experimentally validates the influence of a reduced LWT.^11^ It elaborates further on the understanding of the importance of a medial buttress with a load-sharing implant.^10^ Altogether, it reveals the failure mechanism by overloading the fragile lateral wall in this setting.^4^ To the best of our knowledge, this is the first report evaluating the role of both LWT and lesser trochanter detachment in the formation of lateral wall fractures ex vivo.

The fact that both characteristics were required for lateral wall fractures at a lower loading level questions their internal rank and dependence. In our study, the impact by the lesser trochanter appears more fundamental, affecting failure mechanics and failure patterns in all evaluated osteotomies. Nie et al^6^ examined load to failure in an incomplete trochanteric osteotomy involving a wedge of the lesser trochanter or lateral wall. The lower failure load with the lesser trochanter osteotomy than lateral wall osteotomy in their study (476 N vs 1,597 N, p < 0.001) agrees with our findings of a more important role of medial than lateral stability in this setting. The role of the gap osteotomy extent in this interpretation was emphasized by the study of Marmor et al,^10^ which showed that extending the medial gap proximally to involve the calcar resulted in a gradual increase in implant load-bearing capacity. Regarding the precise location of this upper osteotomy, in our study it was positioned to simulate the avulsion at the iliopsoas tendon attachment. We do not believe that the muscular attachment to the lesser trochanter, which was not modelled in our study, would significantly impact the results, as this attachment plays a more important role in the formation of the avulsion rather than in its stabilization. Noteworthy, Hsu et al^2^ proposed that both characteristics are created by a more distal fracture line, based on the observation of a lower LWT in fractures with a detached lesser trochanter than with an intact one (21 mm vs 30 mm, p < 0.001). If LWT only reflects whether the lesser trochanter is detached, further discussion of rank and dependence on lateral and medial criteria in trochanteric fracture classification may be unnecessary.

Concerning the limitations of our study, ex vivo studies should be interpreted with caution due to the lack of standardized testing protocols. The use of predrilled specimens with anatomical reduction in the current study may not fully reflect the clinical setting, where a lower quality of reduction and a higher TAD raises concerns about a further decrease in stability in the least stable fractures clinically. The absence of soft-tissues and muscular stabilization is a limitation of our model. Although lateral wall fractures often occur perioperatively,^22^ the loading in this study was designed to simulate the direction and magnitude of the hip joint reaction force. Instead of focusing on tensile strength, we prioritized compressive force and employed a rigid displacement constraint to prevent uncontrollable sliding, modelling stumbling as a worst-case scenario. Yet, lateral wall bending tests may be inadequate, only reflecting the settings during surgery. A detailed understanding of the failure mechanism, including strain measurements and high-speed motion capture analysis, would have increased the precision of our study by providing insights into implant load-bearing and stress distribution. Failure defined at 10 mm facilitated our findings of initial failure patterns, where prolonged testing might have unveiled cut-outs secondary to the lateral wall fracture.^3^ Focusing on a fixation-specific complication, and not comparing to other fixation methods, may reduce generalizability of our findings. Lastly, the comparisons may be underpowered in some parameters. However, our significant findings revealed within a small sample size suggest a considerable impact of possible clinical importance. Further studies analyzing the lower fracture lines with comparisons to other fixation methods, such as an intramedullary nail, are needed in this setting.

Despite this, our method may be valid, ensured by a clinically relevant set-up with significant findings. Regarding external validity, difference from relative comparisons of fracture characteristics may withstand, while absolute values may differ from clinical values. However, the simplified osteotomies may not be precise in reflecting the most frequent patterns, but was essential for our stepwise approach and findings.

In conclusion, our findings indicate that both the reduction in LWT and the detachment of the lesser trochanter play critical roles in the failure mechanism of lateral wall fractures, leading to significantly lower load thresholds during trochanteric fracture fixation with a SHS. The initiation of lateral wall fractures requires a medial gap reflective of lesser trochanter detachment, while the importance of reduced LWT is evident in lowering the load required for failure. These results imply a failure mechanism of lateral wall overloading, which is attributed inserting a load-sharing implant through a fragile lateral wall in these unstable fractures.

**Take home message**

- We conducted a biomechanical analysis to investigate the failure mechanics of trochanteric fractures in cadavers fixed with a sliding hip screw.

- Unstable trochanteric fractures, with both detached lesser trochanter and reduced lateral wall thickness, were vulnerable for failure by lateral wall fracture at lower loading.

- The failure mechanism of a lateral wall fracture at low loading is explained by inserting a load-sharing implant in an unstable fracture complex.

## Data Availability

The datasets generated and analyzed in the current study are not publicly available due to data protection regulations. Access to data is limited to the researchers who have obtained permission for data processing. Further inquiries can be made to the corresponding author.
